# Measuring Free-Living Physical Activity With Three Commercially Available Activity Monitors for Telemonitoring Purposes: Validation Study

**DOI:** 10.2196/11489

**Published:** 2019-04-24

**Authors:** Martine JM Breteler, Joris H Janssen, Wilko Spiering, Cor J Kalkman, Wouter W van Solinge, Daan AJ Dohmen

**Affiliations:** 1 Department of Anesthesiology University Medical Center Utrecht Utrecht University Utrecht Netherlands; 2 FocusCura Driebergen-Rijsenburg Netherlands; 3 Department of Vascular Medicine University Medical Center Utrecht Utrecht University Utrecht Netherlands; 4 Department of Clinical Chemistry and Haematology University Medical Center Utrecht Utrecht University Utrecht Netherlands

**Keywords:** activity trackers, telemedicine, exercise

## Abstract

**Background:**

Remote monitoring of physical activity in patients with chronic conditions could be useful to offer care professionals real-time assessment of their patient’s daily activity pattern to adjust appropriate treatment. However, the validity of commercially available activity trackers that can be used for telemonitoring purposes is limited.

**Objective:**

The purpose of this study was to test usability and determine the validity of 3 consumer-level activity trackers as a measure of free-living activity.

**Methods:**

A usability evaluation (study 1) and validation study (study 2) were conducted. In study 1, 10 individuals wore one activity tracker for a period of 30 days and filled in a questionnaire on ease of use and wearability. In study 2, we validated three selected activity trackers (Apple Watch, Misfit Shine, and iHealth Edge) and a fourth pedometer (Yamax Digiwalker) against the reference standard (Actigraph GT3X) in 30 healthy participants for 72 hours. Outcome measures were 95% limits of agreement (LoA) and bias (Bland-Altman analysis). Furthermore, median absolute differences (MAD) were calculated. Correction for bias was estimated and validated using leave-one-out cross validation.

**Results:**

Usability evaluation of study 1 showed that iHealth Edge and Apple Watch were more comfortable to wear as compared with the Misfit Flash. Therefore, the Misfit Flash was replaced by Misfit Shine in study 2. During study 2, the total number of steps of the reference standard was 21,527 (interquartile range, IQR 17,475-24,809). Bias and LoA for number of steps from the Apple Watch and iHealth Edge were 968 (IQR −5478 to 7414) and 2021 (IQR −4994 to 9036) steps. For Misfit Shine and Yamax Digiwalker, bias was −1874 and 2004, both with wide LoA of (13,869 to 10,121) and (−10,932 to 14,940) steps, respectively. The Apple Watch noted the smallest MAD of 7.7% with the Actigraph, whereas the Yamax Digiwalker noted the highest MAD (20.3%). After leave-one-out cross validation, accuracy estimates of MAD of the iHealth Edge and Misfit Shine were within acceptable limits with 10.7% and 11.3%, respectively.

**Conclusions:**

Overall, the Apple Watch and iHealth Edge were positively evaluated after wearing. Validity varied widely between devices, with the Apple Watch being the most accurate and Yamax Digiwalker the least accurate for step count in free-living conditions. The iHealth Edge underestimates number of steps but can be considered reliable for activity monitoring after correction for bias. Misfit Shine overestimated number of steps and cannot be considered suitable for step count because of the low agreement. Future studies should focus on the added value of remotely monitoring activity patterns over time in chronic patients.

## Introduction

### Background

The use of wearable activity trackers has moved into the mainstream, helping people chart their activity behavior over time or see how they perform compared with others. These activity trackers have rapidly evolved from relatively simple mechanical pedometers to sophisticated “connected” activity monitors with built-in accelerometers. Due to this, consumers are increasingly uploading their physical activity record through apps on their mobile phone [1]. Studies have demonstrated that physical activity interventions delivered via internet may result in small increases in activity [2-4].

Activity monitors should not only be used to increase the consumers’ awareness about their physical activity behavior. It is widely known that increased physical activity levels significantly improve health in patients with cardiovascular risk factors such as hypertension or obesity [5-9]. In addition, a substantial body of evidence shows that regular physical activity reduces hospital admissions and mortality in patients with chronic obstructive pulmonary disease (COPD) [10-12]. Measuring activity levels is not only useful to provide patients with personalized feedback, a progressive decrease in activity level could also be an early warning sign for deterioration. For example, patients experiencing a COPD exacerbation may become inactive because of sudden worsening of their lung function [10]. Therefore, daily monitoring of physical activity levels in patients with chronic conditions might be important and could be used as additional measurement in electronic health programs to guide patients via telemonitoring to prevent hospital readmissions or unnecessary outpatient visits.

Simple pedometers have been used in health care for decades. For example, to monitor physical activity or to deliver a step count prescription strategy for patients with type 2 diabetes or hypertension [13,14]. However, these pedometers are difficult to use for remote monitoring, as they require the patient to manually record the daily number of steps and to send these data to the caregiver. On the other hand, consumer activity monitors that can be coupled with mobile apps have the potential to result in positive behavior change [15,16]. At the same time, they offer health professionals real-time assessments of their patients’ daily activity pattern, which allows adjustment of treatment.

Although these new consumer activity trackers offer considerable promise to health care professionals and patients, their adoption into clinical practice is currently limited. Data of activity trackers are rarely integrated into clinical applications for chronic disease management [17]. In part, this is because of the limited evidence regarding validity and reliability of consumer activity trackers, although some studies have examined the reliability of activity trackers in healthy participants as a measure of free-living activity [18-20]. Although a number of studies show the utility of activity monitors to promote behavior change [21], little research is available that shows the potential of using activity trackers in patients with chronic diseases [22,23], which hampers uptake in clinical practice. At the same time, most application studies that involved the use of smart activity monitors lacked any form of usability testing before implementation in health care [24]. In addition, the added value of remote monitoring of physical activity on clinical outcome is unknown. A recent meta-analysis [4] suggested that remote activity programs resulted in small increases in activity in overweight or obese patients. Furthermore, in the ever-growing activity trackers market, it is unclear which consumer activity trackers are sufficiently reliable to be used for remote monitoring rather than for wellness or sports purposes. Most studies evaluated activity trackers under laboratory conditions or controlled environments, such as treadmill walking [25-28]. These results showed moderate-to-good agreement in measuring steps, except for walking at a slower speed. Activity trackers that are validated during free-living conditions were typically worn for a working-day of 8 hours or less, which makes it difficult to draw conclusions about the ability to remotely detect changes in physical activity over time [29].

Given the large number of activity trackers now commercially available, it is important to validate the accuracy and reliability of consumer activity trackers that can be integrated in third-party apps to be able to remotely monitor the patient’s physical activity level. In addition, it is important to evaluate such activity trackers on usability aspects to prevent unnecessary expenses on reliable activity trackers while performance on usability aspects were known inadequate beforehand.

### Objective

The objective of this study was to evaluate the usability and test the validity of 3 commercially available activity monitors as a measure of free-living activity within healthy participants. To investigate which consumer activity trackers are suitable for remote monitoring of physical activity, we used a dual approach in which we first evaluated the usability of activity trackers before testing the validity of the activity trackers in healthy volunteers.

## Methods

### Study Design

The dual approach of this study required the combination of a qualitative and quantitative method. The qualitative part comprised a usability evaluation to explore individuals’ perceptions on different consumer-level activity trackers. The results of this usability evaluation were used to select the activity trackers for validation. The quantitative part comprised a validation study with a comparative design to assess the concurrent validity of 3 consumer activity trackers as measure of physical activity compared with the Actigraph GT9X Link (Actigraph Inc) activity monitor. The Actigraph GT9X Link is considered the gold standard for research grade activity monitoring and has been extensively validated [30,31].

### Study 1: Usability Evaluation

A total of 3 consumer-level activity monitors were chosen on the basis of having a software development kit available that allows their data to be integrated with a third-party app to be used for telemonitoring purposes. The following activity trackers were selected: iHealth Edge (iHealth Labs Inc), Misfit Flash (Misfit Wearables), and Apple Watch (Apple Inc) smartwatch.

A total of 10 adult volunteers were asked to wear one of the selected activity trackers for a period of 30 days. The duration of 30 days was chosen to ensure valuable feedback and prevent integrating activity trackers in third-party apps, which are not perceived as user friendly. At the end of the 30-day period, participants were asked to fill in a questionnaire with open and closed questions about wearability, ease of use, and experiences. These results on usability and experience were discussed afterwards with all participants during a round table discussion. Results of this usability evaluation were used to select the activity trackers for validation. Minimum criteria were a neutral or good score on ease of use and at least a neutral or comfortable score on wearability to proceed to the validation phase. In addition, agreement on the selected activity trackers for validation among the participants of the round table discussion was needed.

### Study 2: Validation Study

The quantitative part comprised a validation study with a comparative design to assess the concurrent validity of 3 consumer activity trackers as measure of number of steps compared with the Actigraph GT9X Link activity monitor. The results from the usability evaluation were used to select the appropriate activity trackers to proceed with the validation study.

In addition to the 3 selected consumer activity trackers, the Yamax Digiwalker SW-200 pedometer was added as the fourth activity tracker to be examined. Although this pedometer cannot be integrated with third-party apps for telemonitoring purposes, we decided to add this pedometer as this is currently being used as standard of care at the outpatient clinic to check whether patients with hypertension exercise 30 min a day. Consequently, a comparison can be made between the accuracy of this pedometer and the 3 activity monitors. All the activity devices used for validation measure various physical activity parameters, except the Yamax Digiwalker (Table 1).

### Recruitment

Participants were eligible to take part if they were aged 18 years or older and reported no known disease or injury that would prevent them from ordinary physical activity for a 72-hour period. Participants were recruited to participate via targeted emails within the Universtity Medical Center Utrecht and by using the individual networks of the researchers. A sample of 30 participants was recruited on the basis of a power calculation with findings of observed step counts (correlation of 0.5), alpha=.05, and a power of 0.80 that indicated a sample size of 28 participants. With an expected dropout rate of 2 participants, it was planned to recruit 30 participants. This is comparable with the sample size used in previous validation studies [32-34]. The study was conducted in accordance with the moral, ethical, and scientific principles as outlined in the Declaration of Helsinki. Formal approval for this study was obtained from the local ethical committee of the University Medical Center Utrecht, the Netherlands (number 16/376).

**Table 1:** Details of activity measurement devices.

Activity measurement devices		Actigraph GT9X Link (reference monitor)	iHealth Edge (activity tracker)	Misfit Shine (activity tracker)	Yamax Digiwalker SW-200 (pedometer)	Apple Watch (smartwatch)
**Parameters measured**						
	Steps	✓	✓	✓	✓	✓
	Distance	x	✓	✓	x	✓
	Intensity levels	✓	x	✓	x	x
	Heart rate	x	x	x	x	✓
	Calories burned	✓	✓	✓	x	✓
	Elevation	x	x	x	x	x
	Sleep time	✓	✓	✓	x	x
	Sleep quality	✓	✓	✓	x	x
Wear site		Right hip (and wrist)	Right hip (and wrist)	Right hip (and wrist)	Right hip (and wrist)	Wrist
Software		Actilife v6.6.3	iHealth MyVitals v	Misfit App v	None	WatchOS

^a^✓: parameter being measured.

^b^x: parameter not being measured.

### Data Collection

All participants were asked to attend an appointment just before the start of the study at which demographic data (gender, date of birth, height, and weight) were obtained and an explanation was given about the study. Participants were asked to wear the Apple Watch on the wrist and all other devices on their waist on the right-hand side. Participants were instructed to leave all devices on simultaneously for a 72-hour period (excluding sleep, swimming, and showering) to capture 3 full days of activity. Compliance was checked using the reference device, which automatically detected nonwear time. The wear period was not restricted to a particular period of the week, and no restrictions on activity were provided to ensure representative data of free-living physical activity. Participants were only advised to be cautious not to lose the devices during extreme activities. Furthermore, they were given an iPhone, which was connected to the activity monitors. As the Yamax Digiwalker cannot distinguish among days, participants were asked to write down the number of steps (read from the display) on a step-log sheet that was provided and reset the pedometer by the end of the day. Data collection took place from June to October 2016.

### Data Handling

Data were cleaned by removing nonwear time for the activity trackers and the Actigraph reference. Before the start of the 72-hour period of each participant, the Actigraph devices were initialized using the manufacturer’s software, ActiLife Version 6.13.2. This software was also used to download all activity data. Data from the Apple Watch were retrieved by importing the xml file, including all data from the Apple Health app. Data from the Misfit Shine were exported using the raw JavaScript Object Notation format. Data from the iHealth Edge were retrieved with comma-separated values’ exports. The update rate differed among activity trackers. The Actigraph and iHealth Edge have a fixed update time of once every minute or once every 5 min, respectively. Both the Apple Watch and Misfit have a variable update rate. The steps per day from the Yamax pedometer were retrieved from the written diary of each participant.

### Statistical Analysis

Statistical analysis was performed using Matlab (The MathWorks, Inc, Version 2016b). The agreement between the consumer level activity monitors and the Actigraph was assessed by calculating the bias (mean difference), the SD, and the 95% limits of agreement (LoA) as described by Bland and Altman [35]. Furthermore, median absolute difference (MAD) compared with the Actigraph was calculated with the following formula: median of |steps activity tracker–steps Actigraph|/steps Actigraph [33]. The MAD was used as data were highly skewed. We considered a MAD ≤15% to be acceptable for clinical purposes [36]. In addition, a correction for potential bias was estimated and validated using the leave-one-out cross validation. Subsequently, the original data were randomly partitioned into *k* (with *k*=30) subsamples. Of the 30 subsamples, k-1 subsamples were used as training data, and the remaining subsample was used as test data. The cross-validation was repeated k times, each time leaving out a different pair to use as the single test data. Of the predicted estimate, the MAD was calculated and evaluated.

## Results

### Usability Evaluation

A total of 10 volunteers participated in the usability evaluation for a period of 30 days each. A total of 2 volunteers wore the iHealth Edge, whereas Misfit Flash and Apple Watch each were tested by 4 volunteers. iHealth Edge and Apple Watch were described as comfortable or very comfortable to wear (see Tables 2 and 3). The wearability of the Misfit Flash was perceived as uncomfortable or neutral by 2 out of 4 participants. Moreover, this activity tracker broke down or was lost in 2 cases. Participants reported the following:

The fixation was so unstable that I unfortunately lost it.

The lifetime of the waist clip is very short.

No complaints were mentioned regarding the other activity trackers; however, 2 volunteers reported that they needed a little more time to understand the basic working principles of the Apple Watch. One volunteer reported that tapping on the iHealth screen did not always show the number of steps immediately.

On the basis of these results, the Misfit Flash was replaced by the more sustainable Misfit Shine during the subsequent validation study.

### Study Population

A total of 30 volunteers participated in the quantitative study. Gender distribution was approximately equal with 14 females and 16 males, with age ranging from 23 to 58 years (mean age 40.4, SD 10.6 years) and body mass index (BMI) ranging from 18.8 to 36.6 kg/m^2^—median BMI 23.8 (21.8-25.7).

All 30 participants wore the full set of 3 activity trackers, the pedometer, and reference for a 72-hour period. However, some data were lost because of device malfunctioning (7 days for the Yamax Digiwalker and 3 days for the iHealth Edge), participant error (2 days for Misfit Shine because of losing the activity tracker), empty battery in 3 participants for the Apple Watch, or data extraction error (9 sets of Misfit Shine data). A total of 11 hours were missing for the reference standard in 2 participants. Nonwear time of the reference monitor was analyzed and corrected in all data pairs. Empty or invalid data (“not-a-number”) were removed and excluded for analysis. Number of data points received varied considerably among activity trackers. On average, over a period of 72 hours per participant, 4300 data points were received from Actigraph, 722 data points from iHealth Edge, 467 data points from Apple Watch, 33 data points from Misfit Shine, and 2.8 data points from the Yamax Digiwalker. No data points were received during nighttime and showering.

**Table 2:** Usability evaluation activity trackers in general. For these “general questions” we used the information of all participants asked during the usability part (N=10).

Question		General (N=10)
**What is your preferred location to wear an activity tracker?**		
	Wrist	7
	Waist	2
	On the shoe	1
**Have you worn an activity tracker before?**		
	Yes	6
	No	4
**In general, how have you perceived wearing an activity tracker?**		
	Very bad	0
	Bad	2
	Not good, not bad	3
	Good	4
	Very good	1
**How important is the look and feel of an activity tracker?**		
	Not important	0
	Slightly important	2
	Moderately important	1
	Important	7
	Very important	0
**On which device would you like to see your activity records?**		
	Phone	10
	Tablet	2
	Computer	1

**Table 3:** Usability evaluation of the activity trackers.

Question		Total (N=10)	iHealth Edge (N=2)	Apple Watch (N=4)	Misfit Flash (N=4)
**What is the ease of use?**					
	Very difficult	0	0	0	0
	Difficult	0	0	0	0
	Neutral	2	1	0	1
	Easy	7	1	3	3
	Very easy	1	0	1	0
**How did you perceive the wearability?**					
	Very uncomfortable	0	0	0	0
	Uncomfortable	1	0	0	1
	Neutral	1	0	0	1
	Comfortable	6	2	2	2
	Very comfortable	2	0	2	0
**To what extent stimulated the activity tracker to move more?**					
	Not at all	2	0	0	2
	To some extent	2	1	0	1
	To a moderate extent	3	1	2	0
	To a great extent	2	0	1	1
	To a very great extent	1	0	1	0

### Level of Agreement

Activity as measured with the reference device varied between 10,757 and 35,818 steps (median: 21,527 steps, IQR 17,475-24,809). Bias and precision (95% LoA) from comparisons between the activity trackers and the reference standard are shown in Table 4. The mean difference (bias) in steps was 968 between the Actigraph and Apple Watch, with a 95% LoA of −5478 to 7414 steps over a 72-hour period. The iHealth Edge showed a mean difference of 2021 and a 95% LoA of −4994 to 9036 steps. The Misfit Shine showed a mean difference of −1874 steps with wide limits levels of agreement (95% LoA: −13,869 to 10,121 steps). The mean difference of the Yamax Digiwalker compared with the Actigraph was on average 2004, also with wide levels of agreement (95% LoA of −10,932 to 14,940 steps).

**Figure 1:**
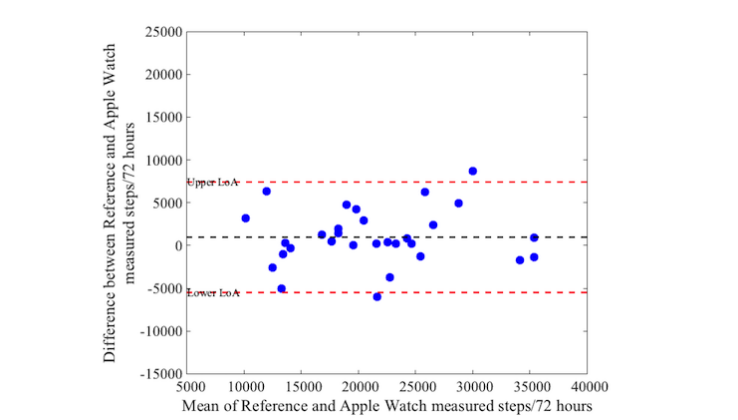
Bland-Altman plot for Apple Watch versus Actigraph step counts over a 72 hour period (n=30).

**Figure 2:**
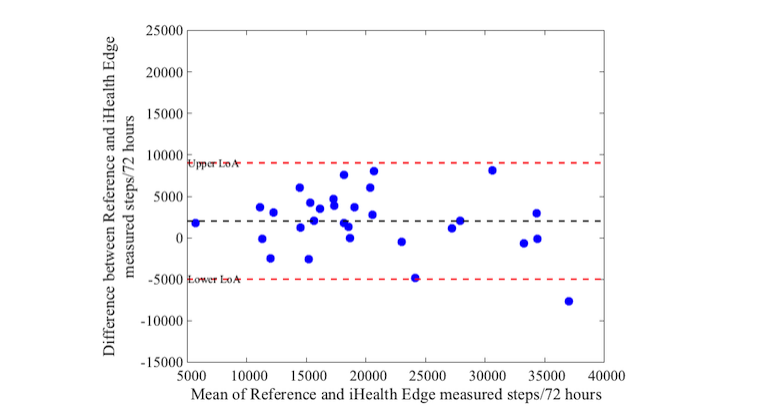
Bland-Altman plot for iHealth Edge versus Actigraph steps over a 72 hour period (n=30).

**Figure 3:**
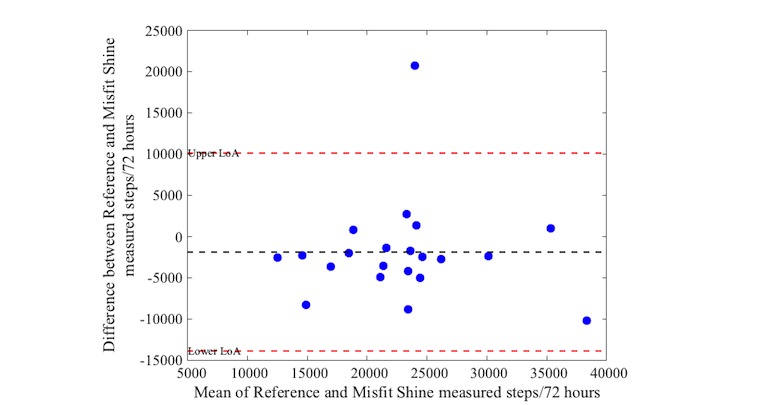
Bland-Altman plot for Misfit Shine versus Actigraph steps over a 72 hour period (n=21).

**Figure 4:**
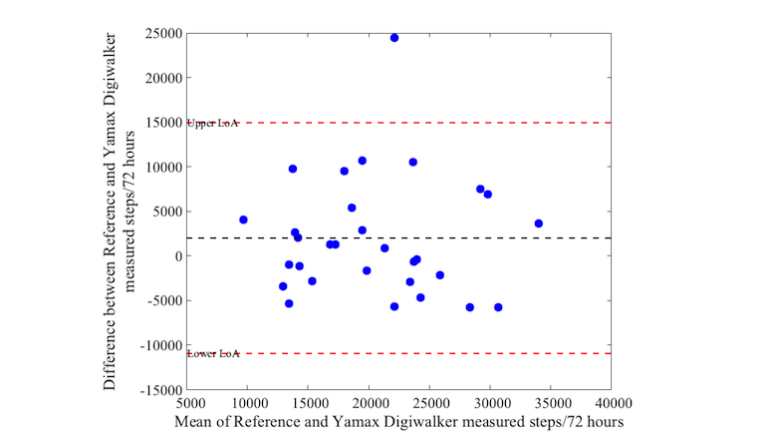
Bland-Altman plot for Yamax Digiwalker versus Actigraph steps over a 72 hour period (n=30).

Figures 1 to 4 illustrate the Bland-Altman plots of all 4 activity trackers. The Apple Watch (Figure 1) and iHealth Edge (Figure 2) showed the narrowest LoA, whereas the Misfit Shine and Yamax Digiwalker both showed wide LoA (Figures 3 and 4).

**Table 4:** Bland-Altman analysis for the activity measurement devices versus the reference monitor.

Activity measurement devices	MAD^a^ (%)	SD	Bias^b^	Lower 95% LoA^c^	Upper 95% LoA	MAD (% after correction)^d^
Apple Watch	7,7	3289	968	−5478	7414	10.3
iHealth Edge	19,0	3579	2021	−4994	9036	10,7
Yamax Digiwalker	20,3	6600	2004	−10,932	14,940	21.6
Misfit Shine	16,7	6120	−1874	−13,869	10,121	11.3

^a^MAD: median absolute differences.

^b^A positive bias indicates an underestimation of number of steps, whereas a negative bias means an overestimation.

^c^LoA: limits of agreement.

^d^Accuracy estimate using leave-one-out cross validation.

### Median Absolute Difference

**Figure 5:**
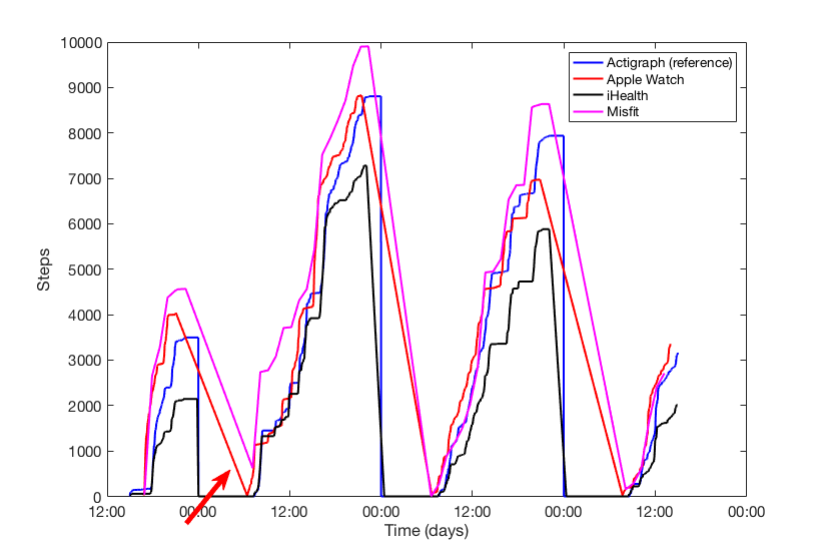
Example of the number of steps over time of the different activity trackers within one participant. The red arrow is pointed towards the number of steps of Misfit Shine that seems to add number of steps overnight or at the beginning of a day.

The MAD percentages are shown in Table 3. The Apple Watch noted the smallest MAD of 7.7% with the Actigraph. The MAD of the iHealth Edge, Yamax Digiwalker, and Misfit Shine were higher than the accepted clinical difference of 15% (19.0%, 20.3%, and 16.7%, respectively). Note that the number of steps of the Misfit Shine (SD 6120) and Yamax Digiwalker (SD 6600) was highly variable when compared with the iHealth Edge (SD 3579) and Apple Watch (SD 3289). After correction for potential bias using leave-one-out cross validation, the accuracy estimates for MAD improved for the iHealth Edge (10.7%) and Misfit Shine (11.3%). Figure 5 shows an example of the number of steps of all activity trackers of 1 participant. Although the MAD of the Misfit Shine compared with the Actigraph after leave-one-out cross validation may be clinically acceptable, it seems to add number of steps (ie, not reset to 0 completely) during the first night. After inspection, this pattern was seen in 13 participants.

## Discussion

### Principal Findings

In this study, we investigated the performance of different activity trackers intended for potential use in clinical monitoring applications. In 2 studies, we evaluated the usability of different consumer-level activity monitors and subsequently validated selected activity monitors in adult healthy volunteers during free-living conditions. Usability evaluation showed inferior usability of the Misfit Flash activity tracker and better usability of the iHealth Edge and Apple Watch. The validation study showed that the performance of activity monitors varied considerably. The Apple Watch can accurately measure number of steps with a deviation within 15% of the reference standard. In contrast, the accuracy of both Misfit Shine and Yamax Digiwalker was outside the limits we considered acceptable. The mean difference of the iHealth Edge with the Actigraph was high and showed substantial underestimation of steps. After correction for bias using leave-one-out cross validation, accuracy of the iHealth Edge was within acceptable limits. The accuracy of Yamax Digiwalker did not improve after correction for bias, and it was therefore least accurate as compared with the activity trackers with accelerometer. Furthermore, the wide level of agreement and the inability to measure the number of steps more frequent during the day makes this conventional pedometer unsuitable for step count monitoring.

Although the mean difference of the Misfit Shine with the Actigraph was acceptable after leave-one-out cross validation, it cannot be considered suitable for step count monitoring because of the low agreement. In addition, Misfit Shine seems to add number of steps overnight or at the beginning of a day (ie, not completely reset to 0) or at the beginning of a day. We do now know what caused this phenomenon but speculate that it may be related to the low frequency of data transmission from the activity tracker to the mobile phone (approximately once every 2 hours).

Physical activity monitors that allow patients to upload their data within a clinical telemonitoring app may offer significant advantages over traditional pedometers, as it helps doctors remotely to assess their patients’ activity pattern over time and inform treatment [37]. To rely on such activity monitors, it is important to be able to recognize trend patterns in activity over time. Given the results on accuracy and usability in this study, the use of the Apple Watch seems a suitable device to measure physical activity for telemonitoring. Although the MAD of the Misfit Shine improved after leave-one-out cross validation, the number of steps is highly variable. Therefore, a low MAD may not necessarily indicate a high reliability as can be seen in Figure 5. Furthermore, it should be noted that using the iHealth Edge for telemonitoring purposes may disappoint or discourage patients who try to reach their daily activity goals because of the underestimation in steps. However, one might decide to correct for the systematic underestimation.

The results of this study show that not all consumer activity trackers are reliable enough for use in telemonitoring purposes. Both the Apple Watch and iHealth Edge could be used to integrate in third-party apps for personalized care of chronic patients. However, health benefits are only achieved when patients engage in using activity trackers for a prolonged time. A recent yearlong study showed that the use of the Fitbit Zip barely motivated people to move more over time [38]. After the incentive period with cash stopped at 6 months, compliance to wear the activity tracker dropped further from 90% to 40% at 12 months. This suggests that it may be difficult to persuade patients to use the activity tracker for a long time. However, this is difficult to conclude, as cash incentives may work different on intrinsic behavior [39]. At this point, a smartwatch such as the Apple Watch may favor prolonged use, as it returns individualized feedback on the basis of monitoring.

### Limitations

This study has some limitations. First, it should be noted that this study was conducted in healthy participants and not in chronic patients who may have different activity patterns, although it is also known that reliability of activity trackers is reduced at slower walking speed [28,40,41]. In addition, participants were not asked to annotate their variety of different activities from low to more intensive exercise. Consequently, activity trackers may perform differently during different exercise levels. The advantage of this method was that we could identify the reliability of the trackers during free-living conditions, which is more realistic than within a controlled environment. The latter is important when remote monitoring of physical activity levels is being used for telemonitoring purposes.

In this study design, both a usability evaluation and a validation study were performed. Although the combination of a qualitative and quantitative approach is a strength of this study, the number of volunteers who evaluated each of the activity trackers on usability was limited. However, it was assumed that user experience needed to be sufficient before proceeding to the validation phase. Without this usability evaluation, other activity monitors would have been validated, which potentially will not be used for remote monitoring. Conducting both studies separately would therefore not direct to the conclusions as provided in this study.

A third limitation was the use of only step counts as outcome measure of physical activity. The number of steps may not give an adequate picture regarding the amount of physical activity; therefore, intensity of movements and the amount of time spent in different intensities would be more effective to use. Nevertheless, remote monitoring of the number of steps over time may still recognize a changing trend in activity. For example, inactivity in patients with COPD may be recognized as a manifestation of disease severity or the onset of an exacerbation [10]. Another limitation was that the results of the activity trackers were compared with fewer number of Misfit Shine measurements because of errors in extraction of the data. However, the weak correlation of Misfit with the reference monitor and the highly variable mean differences in steps over time make more accurate results for a larger dataset of Misfit measurements unlikely.

### Comparison With Previous Work

The design of this study is unique; therefore, other studies that confirm our findings are limited. Most of the research studies have used Fitbit devices and found preliminary evidence for validity in measuring steps, although some of the results have recorded significant higher number of steps during free-living conditions [18,19,33]. These differences could arise from differences in instrument sensitivity thresholds of devices or because of differences in attachment while wearing the activity tracker [42]. The high variety in number of steps measured over 72 hours among subjects and among devices may be explained by the longer measurement period or could be a result of more nonwalking activities. The few studies that investigated the Misfit Shine all reported good agreement with the reference device in contrast to the findings of this study [29,33,43,44]. This may be explained by the different measurement setups, which was either a controlled environment where participants were instructed to walk repeated sets of 200, 500, or 1000 steps [43], or the analysis included only a maximum of 1 full calendar day or 1 working day, and as such, this may not fully represent free-living conditions [33,36]. As found in this study, the study by Farina et al [44] also found an overestimation of the number of steps with the waist-worn Misfit Shine; however, they found good agreement as compared with the reference. The main difference with this study is that healthy community dwelling older adults with a mean age of 72.5 years were asked to participate, which is much higher as compared with the mean age of this study (40.4 years).

Although it is known that Apple Inc collected more data on activity with the Apple Watch in their exercise lab, results within literature are scarce. One study showed high accuracy (>99.1%) for step count, but these results were obtained under controlled walking conditions [43]. Although the Actigraph is the most commonly used reference standard during research studies, another study showed weak-to-moderate accuracy of the Actigraph during slower speeds (3.2 and 4.0 km/h^-1^) when compared with manually counted steps [34]. It is likely that participants in this study may have been active with lower speeds as well, as most of them also wore the activity trackers during working hours where a slower speed is common. A recent study showed that the mean relative error of the iHealth Edge increases when speed decreased in healthy adults during laboratory conditions [45]. However, the iHealth Edge has not been studied within free-living conditions to confirm the findings of this study. The Yamax Digiwalker used to be one of the best pedometers with regard to accuracy for counting steps years ago [26,46], but the accuracy of a pedometer highly depends on the placement on the waist. The other activity monitors investigated in this study contain microelectromechanical system accelerometers, which can track acceleration in 3 dimensions, and therefore provide increased sensitivity. In this study, no steps were counted in 7 days, possibly because of wrong placement. Furthermore, the accuracy on measuring the number of steps was least accurate as compared with the activity monitors with accelerometers. Beside this, the number of steps can only be manually collected once a day. Therefore, the Yamax Digiwalker is not suitable for objectively measuring the number of steps.

The findings of this study extend the previous research by indicating that the Apple Watch and iHealth Edge can accurately measure steps in-free living conditions over longer durations. As opposed to previous studies, none of the research performed show results on the consistency and variability of steps measured over time as compared with the results as shown in Figure 5 of this study. Furthermore, none of the studies showed results of coupled activity trackers within third-party apps for remote monitoring. Future research should use a more comprehensive framework to study a variety of usability aspects of patients who interact with activity trackers. We also suggest that future studies should integrate validated activity trackers within clinical apps to become part of the treatment “prescription” of health care professionals for remote monitoring of patients.

### Conclusions

The Apple Watch is usable and reliable for activity monitoring within healthy participants. The iHealth Edge underestimates number of steps, but it can be considered reliable for activity monitoring after correction for bias. Misfit Shine overestimated number of steps and cannot be considered reliable because of high variability. The Yamax Digiwalker pedometer performed least accurately for step count and is not reliable to indicate number of steps per day. Both Apple Watch and the iHealth Edge show the potential to be integrated within clinical apps for tracking activity patterns over time. Future studies should focus on the added value of monitoring activity trend patterns within chronic patients, whether or not in combination with other vital signs measured remotely.

## Abbreviations

BMI: body mass index
COPD: chronic obstructive pulmonary disease
IQR: interquartile range
LoA: limits of agreement
MAD: median absolute differences
